# Influence of a 30-Day Slow-Paced Breathing Intervention Compared to Social Media Use on Subjective Sleep Quality and Cardiac Vagal Activity

**DOI:** 10.3390/jcm8020193

**Published:** 2019-02-06

**Authors:** Sylvain Laborde, Thomas Hosang, Emma Mosley, Fabrice Dosseville

**Affiliations:** 1 Department of Performance Psychology, German Sport University Cologne, Institute of Psychology, 50933 Cologne, Germany; 2 Université de Caen Normandie-UFR STAPS, EA 4260 Caen, France; fabrice.dosseville@unicaen.fr; 3 Department of Psychology, Helmut Schmidt University, 22043 Hambourg, Germany; hosang@hsu-hh.de; 4 University of the Federal Armed Forces Hambourg, 22043 Hamburg, Germany; 5 Solent University Southampton, Southampton SO14 0YN, UK; emma.mosley@solent.ac.uk

**Keywords:** parasympathetic nervous system, cardiac vagal tone, high-frequency heart rate variability (HF-HRV), deep breathing, slow breathing, cardiac coherence, vagus nerve, respiratory sinus arrhythmia, vagal tank theory, neurovisceral integration model

## Abstract

Breathing techniques are part of traditional relaxation methods; however, their influence on psychophysiological variables related to sleep is still unclear. Consequently, the aim of this paper was to investigate the influence of a 30-day slow-paced breathing intervention compared to social media use on subjective sleep quality and cardiac vagal activity (CVA, operationalized via high-frequency heart rate variability). Healthy participants (*n* = 64, 33 male, 31 female, *M* = 22.11, *SD* = 3.12) were randomly allocated to an experimental or control group. In the experimental group, they had to perform slow-paced breathing for 15 min each evening across a 30-day period. This was administered through a smartphone application. The control group used social media (e.g., Facebook, Instagram, Whatsapp) for the same duration. The night before and after the intervention, their CVA was assessed via a light portable Electrocardiogram (ECG) device, and they had to fill out the Pittsburgh Sleep Quality Index questionnaire. Results showed that in comparison to the use of social media, the slow-paced breathing technique improved subjective sleep quality and increased overnight CVA, while a tendency was observed for morning awakening CVA. Slow-paced breathing appears a promising cost-effective technique to improve subjective sleep quality and cardiovascular function during sleep in young healthy individuals.

## 1. Introduction

Issues with sleep are a pressing concern for individuals, given they directly impact life quality and represent a risk factor at several levels [1]. Breathing techniques are part of traditional methods used to improve sleep [2]; however, their influence on psychophysiological variables related to sleep is still unclear. This paper is aimed to investigate the influence of a slow-paced breathing intervention on subjective sleep quality and on a psychophysiological variable linked to relaxation states, cardiac vagal activity (CVA), which reflects the activity of the vagus nerve regulating cardiac functioning [3,4,5].

One of the main hypothesis regarding the cause of sleep disturbances is that they may be associated with a state of hyperarousal [6,7]. Methods aiming to decrease a state of hyperarousal usually target an activation of the parasympathetic nervous system, and more specifically of its main nerve, the vagus nerve [8,9]. One way to do so is to use slow-paced breathing [10,11,12,13]. 

Spontaneously, most people breathe between 12 and 20 cycles per minute [14,15]. Slow-paced breathing refers to the act of voluntarily slowing down the breathing rate to a frequency close to 6 cycles per minute (cpm) [12]. The term “paced” means that participants have to follow a visual, auditory, or kinesthetics pacer regulating the duration of inhalation and exhalation phases (for example see [16,17]). Importantly, exhalation should last slightly longer than inhalation, provoking a higher increase of CVA due to the activation of the vagus nerve during exhalation [18,19]. According to the resonance frequency model [12,20], four processes help to explain the positive effects of performing slow-paced breathing at 6 cpm: (1) the phase relationship between heart rate oscillations and breathing at 6 cpm; (2) the phase relationship between heart rate and blood pressure oscillations at 6 cpm; (3) the activity of the baroreflex; and (4) the resonance characteristics of the cardiovascular system. Combined, those processes are expected to strengthen homeostasis in the baroreceptor [21,22,23], which results in improving gas exchanges at the level of alveoli and in increasing vagal afferences [12,20]. 

Slow-paced breathing is assumed to increase the activity of the afferent branch of the vagus nerve [12,20]. Although it is not possible to non-invasively measure the afferent activity of the vagus nerve, there is a way to operationalize non-invasively the efferent activity of the vagus nerve, and more specifically the activity of the vagus nerve regulating cardiac functioning (i.e., CVA) via heart rate variability [3,4,5,24,25]. Dozens of parameters can be extracted from heart rate variability analysis; however, only a handful have been found to reflect CVA, and in the current study we operationalize CVA via one of its most common indicators: high-frequency heart rate variability [3,4,5,24,25]. 

CVA represents the core of several theories (for a summary, see [3]), such as the neurovisceral integration model [26], the polyvagal theory [27], or the more recent vagal tank theory [28]. Taken together, those theories highlight the role of CVA in phenomena such as emotion and stress regulation, executive cognitive performance, social functioning, and health. In order to better understand CVA functioning, it is important to consider several levels of functioning: resting, reactivity, and recovery [3,28]. Further, CVA can be influenced by many factors [29,30], and slow-paced breathing is a straightforward method to increase CVA resting levels [12,16,31].

The question whether measuring CVA during sleep (CVA_night_) represents an indicator of sleep quality is still debated. Some evidence points toward an association between lower CVA_night_ and sleep disorders [32], such as chronic fatigue [33] and insomnia [34]. Higher CVA_night_ has also been related to higher subjective sleep quality [34,35,36]. However, some authors argue that measuring CVA_night_ across sleep stages does not provide useful information given the variations observed in CVA during different sleep stages [37], namely CVA withdrawal during rapid eye movement (REM) sleep, and CVA increase during non-REM sleep [38]. Further, Werner and colleagues [37] argue that assessing CVA while sleeping is suboptimal, given CVA is supposed to reflect adaptations to environmental changes, and these generally do not occur during the night. Thus, they rather recommend assessing CVA during periods where individuals are awake. In summary, even if CVA_night_ measurement cannot be considered as an index of sleep quality, it may still provide an indication of the restorative status of the body during sleep, given it indexes the activity of the parasympathetic nervous system [3,4,15].

In order to address the criticisms made to CVA_night_ measurements, authors have suggested to measure CVA during wake periods [37]. Particularly, a quiet awakening morning period (CVA_morning_) has been suggested as a good compromise, given the individual has usually not experienced heavy environmental changes beforehand [39]. CVA_morning_ has already been related to subjective indices of well-being and to physical training adaptations (see for example [39,40]), and also more recently to subjective sleep quality measurements [41]. Investigating CVA_morning_ together with CVA_night_ measurements seems therefore an appropriate combination to further understand the effects of slow-paced breathing on CVA. 

To the best of our knowledge, only one previous study investigated the effects of slow-paced breathing on sleep [42]. This study, focusing on self-reported insomniacs, aimed to investigate whether a 20-min slow-paced breathing session (6 cpm), compared to a control condition with paced breathing set at 12 cpm, would enhance objective sleep quality as assessed via polysomnography and CVA. In the slow-paced breathing condition, the inhalation and exhalation phases were set to 3 s and 7 s, while no indications were mentioned regarding the inhalation and exhalation phases for the 12 cpm breathing condition. In regards to polysomnography, results showed that after a single 6 cpm session before going to sleep, sleep onset latency, number of awakenings, and awakening time during sleep were decreased, while sleep efficiency was increased, in comparison to the 12 cpm breathing condition and to baseline. Regarding CVA, the heart rate variability variables mentioned in the paper (total power and R-R intervals) unfortunately do not reflect it [3,4,5,24,25]; therefore, it is not possible to draw any conclusions related to CVA. Moreover, heart rate variability was not assessed during sleep, but during daytime rest. Consequently, further studies are therefore warranted to better understand the effects of slow-paced breathing on subjective sleep quality and CVA, and not only on a short-term single session basis, but also on a long-term intervention basis.

In summary, the current study aims to address previous research gaps, investigating the effects of a 30-day slow-paced breathing intervention on subjective sleep quality and CVA_night_ and CVA_morning_. Based on previous research [16,31] and on the resonance frequency model [12], we hypothesize that in comparison to a control condition involving spontaneous breathing, a 30-day slow-paced breathing intervention would increase subjective sleep quality as well as CVA_night_ and CVA_morning_. Finally, due to contradictory evidence [34,35,36,37], no hypothesis was formulated regarding the relationship between subjective sleep quality with CVA_night_ and CVA_morning_.

## 2. Materials and Methods

### 2.1. Participants

We recruited 70 participants, randomly allocated to the experimental group or to the control group. Due to technical problems (*n* = 2) and inability to realize the complete intervention protocol for personal reasons (*n* = 4), the data of 64 participants (33 male, 31 female, *M* = 22.11, *SD* = 3.12, age range = 18–29 years old) were analysed. The body mass index (BMI) of participants was in the normal range, from 18.5 to <25 kg/m^2^. In order to meet the inclusion criteria for the study, participants had to be non-smokers, and not be suffering from sleep disorders (score lower than 5 on the Pittsburgh Sleep Quality Index) or from cardiovascular diseases (self-reported). All participants gave their informed consent for inclusion before they participated to the study. The study was conducted following the Declaration of Helsinki, and the protocol was approved by the Ethics committee of the German Sport University Cologne (Project identification code 42/2015).

### 2.2. Measures

#### 2.2.1. Subjective Sleep Quality—Pittsburgh Sleep Quality Index

In order to measure subjective sleep quality, the German version [43] of the Pittsburgh Sleep Quality Index (PSQI; [44]) was used. This self-report questionnaire assesses sleep quality for the four preceding weeks. A total of 18 items serve to generate seven component scores (which values are comprised between 0 and 3): subjective sleep quality, sleep latency, sleep duration, habitual sleep efficiency, sleep disturbances, use of sleeping medication, and daytime dysfunction. A global measure of subjective sleep quality, ranging from 0 to 21, is then calculated, with lower values indicating better sleep quality (Table 1).

#### 2.2.2. Cardiac Vagal Activity (Operationalized via High-Frequency Heart Rate Variability)

In this study, CVA was operationalized via high-frequency heart rate variability (0.15–0.40 Hz) absolute power calculated via fast Fourier transform [3,4,5]. Additionally, as suggested by Laborde et al. [3], additional heart rate variability variables are presented in the descriptive statistics in Table 2, and the full data set is uploaded as Supplementary Materials. An ECG device (Faros 180°, Mega Electronics, Kuopio, Finland) was used during the experiment to assess heart rate variability, with a sampling rate of 500 Hz. We used two disposable ECG pre-gelled electrodes (Ambu L-00-S/25, Ambu GmbH, Bad Nauheim, Germany). The negative electrode was placed in the right infraclavicular fossa (just below the right clavicle) while the positive electrode was placed on the left side of the chest, below the pectoral muscle in the left anterior axillary line. From ECG recordings we extracted the heart rate variability variables with Kubios© (University of Eastern Finland, Kuopio, Finland). The full ECG recording was inspected visually, and artefacts were corrected manually [3]. Short-term morning measurements followed the five-minute duration recommendation [3,4], while overnight measurements were calculated from the time spent in bed (self-reported by the participants). As recommended by Laborde et al. [3], respiratory frequency was also assessed. In the current study respiratory frequency was computed via the ECG-derived respiration algorithm of Kubios© [45].

### 2.3. Intervention

#### 2.3.1. Experimental Group: Slow-Paced Breathing

Participants in the experimental group had to realize the slow-paced breathing technique for 15 min before going to sleep, using the smartphone app “Breath Pacer”, displaying a flower slowly adding petals to indicate inhalation (4.5 s) and exhalation (5.5 s) phases. Participants had to inhale via the nose, and exhale via pursed lips. The respiratory pattern was based on previous research investigating the influence of slow-paced breathing on psychophysiological outcomes [16,17].

#### 2.3.2. Control Group: Social Media Use

Participants in the control group had to use social media (e.g., Facebook, Instagram, Whatsapp) for 15 min before going to sleep, in order to mirror a typical smartphone use with spontaneous breathing. They were given no specific instructions related to breathing patterns.

### 2.4. Procedure

Participants were recruited via flyers at the local University. They were asked to come to the lab for a presentation of the experiment (Day 1), and they were allocated randomly to either the experimental group (slow-paced breathing) or to the control group (social media use). They were told that the study was about investigating the effects of a smartphone-based relaxation method on heart rate during sleep. Participants in the experimental group were given an introduction to the slow-paced breathing technique and were performing it for 15 min together with the experimenter, ensuring that they understood correctly how to perform it at home. Participants in the control group were given an introduction for the same duration about the relaxing effects of social media. All participants also filled out the PSQI and the demographic questionnaire related to heart rate variability from Laborde et al. [3]. Participants had to come back to the lab at Day 2 between 16:00 and 20:00 h in order to get the ECG device and electrodes attached. This day had to be a weekday, to have participants following their usual daily activities and sleeping routines. They were told that the last meal should be taken at least 2 h before going to bed, and that afterwards only drinking water was allowed. They were also not allowed to drink alcohol or have strenuous physical activity on this day or the day before. 

Participants were asked to have a similar day structure for both evaluation days (pre-test and post-test), in order to provide the best comparison possible for heart rate variability measurements. No intervention was performed in the evening preceding the pre-test and post-test night measurements. Participants were told to start the device before going to bed, write down the time when they went to bed, and then turn off the light. We are aware that lying in bed does not imply that participants were sleeping, and that sleep onset latency may differ across participants, but this variable has been assessed in previous studies [42] and represents a compromise when polysomnography assessment is not available. In the morning participants had to write down waking time, and stay in bed for 5 min after awakening for the morning awakening heart rate variability measurement. During the 30-day intervention participants had to confirm every night via an online form that they either did the slow-paced breathing exercise for the experimental group or that they used social media for the control group. In case participants forgot to do so, they were gently reminded by a research assistant on the next day to continue with the procedure. The maximum of misses was set to three (10%); more than three misses was then considered as a dropout. At the end of the intervention, participants were coming back to the lab to get the ECG device for the post-test and filled out again the PSQI. The post-test night and morning measurements followed the same procedure as for the pre-test, and participants were asked to follow the same daily routine, as well as having the same times to go to bed and to wake up. The next day the participants brought the device back to the lab, and they were debriefed about the aim of the experiment. For the control group, the debriefing included an introduction to the slow-paced breathing technique.

### 2.5. Data Analysis

Data analysis was realized with JASP (Version 0.9.2, JASP Team, 2018). Data was checked for outliers and normal distribution. Outliers (±3.27 SD) were winsorized (3.3%). Given the heart rate variability data was not normally distributed it was log transformed (log10), as recommended by Laborde et al. [3]. Repeated-measures ANOVAs were conducted with time (pre-test vs. post-test) as a within-subject variable, and with condition (experimental vs. control) as between-subject variable. Dependent variables were time spent in bed, PSQI, CVA_night_, breathing frequency_night_ night, CVA_morning_, and breathing frequency_morning_. When interaction effects were found, we calculated four additional Student’s *t*-tests to investigate the interaction effects, and therefore for the post-hoc tests we adjusted the alpha level via Bonferroni correction to 0.05/4 = 0.0125. Finally, two Pearson correlations were run between PSQI change and CVA_night_ change and between PSQI change and CVA_morning_ change, with a significant threshold set to 0.5/2 = 0.025. The change was obtained subtracting the pre-test value from the post-test value. Effect sizes are indicated via partial *η*^2^ and Cohen’s *d*.

## 3. Results

Regarding the time spent in bed, a repeated-measure ANOVA revealed no main effect of time, *F* (1,62) = 1.808, *p* = 0.184, partial *η*^2^ = 0.03, and no time × condition interaction effect, *F*(1,62) = 0.651, *p* = 0.423, partial *η*^2^ = 0.01.

### 3.1. PSQI

A repeated-measure ANOVA revealed no main effect of time, *F* (1,62) = 0.070, *p* = 0.685, partial *η*^2^ = 0.01. An interaction effect time × condition was found, *F* (1,62) = 9.744, *p* = 0.003, partial *η*^2^ = 0.14. Concerning simple main effects for condition, there was no significant difference between the conditions at pre-test, *t*(62) = 0.449, *p* = 0.655, *d* = 0.11, but there was a tendency for a difference between the conditions at post-test (i.e., PSQI score lower for the experimental group in comparison to the control group), *t*(62) = 2.481, *p* = 0.016, *d* = 0.62. Concerning simple main effects for time, there was a significant difference between pre-test and post-test for the experimental group (i.e., PSQI score decrease, indicating higher subjective sleep quality), *t*(31) = 2.881, *p* = 0.007, *d* = 0.51, but no significant difference was found for the control group, *t*(31) = 1.717, *p* = 0.096, *d* = 0.30.

### 3.2. CVA_night_

A repeated-measure ANOVA revealed a tendency for a main effect of time, *F*(1,62) = 3.967, *p* = 0.051, partial *η*^2^ = 0.05. An interaction effect time x condition was found, *F*(1,62) = 16.449, *p* < 0.001, partial *η*^2^ = 0.20. Concerning simple main effects for condition, there was no difference between the conditions at pre-test, *t*(62) = 0.793, *p* = 0.411, *d* = 0.20 nor at post-test *t*(62) = 1.383, *p* = 0.172, *d* = 0.35. Concerning simple main effects for time, there was a significant difference between pre-test and post-test for the experimental group (i.e., CVA_night_ increase), *t*(31) = 3.868, *p* < 0.001, *d* = 0.68, but no significant difference was found for the control group, *t*(31) = 1.655, *p* = 0.108, *d* = 0.29.

### 3.3. Breathing Frequency_night_

A repeated-measure ANOVA revealed no main effect of time, *F*(1,62) = 0.065, *p* = 0.800, partial *η*^2^ = 0. A significant interaction effect time × condition was found, *F*(1,62) = 4.279, *p* = 0.043, partial *η*^2^ = 0.06. Concerning simple main effects for condition, there was no difference between the conditions at pre-test, *t*(62) = 0.296, *p* = 0.296, *d* = 0.26 nor at post-test *t*(62) = 0.059, *p* = 0.953, *d* = 0.02. Concerning simple main effects for time, there was no significant difference between pre-test and post-test for the experimental group, *t*(31) = 1.500, *p* = 0.004, *d* = 0.27 or for the control group, *t*(31) = 1.433, *p* = 0.162, *d* = 0.25.

### 3.4. CVA_morning_

A repeated-measure ANOVA revealed no main effect of time, *F*(1,62) = 0.360, *p* = 0.551, partial *η*^2^ = 0.01. An interaction effect time × condition was found, *F*(1,62) = 6.533, *p* = 0.013, partial *η*^2^ = 0.10. Concerning simple main effects for condition, there was no difference between the conditions at pre-test, *t*(62) = 1.001, *p* = 0.321, *d* = 0.25 nor at post-test *t*(62) = 1.163, *p* = 0.249, *d* = 0.29. Concerning simple main effects for time, there was a tendency for a difference between pre-test and post-test for the experimental group (i.e., CVA_morning_ increase), *t*(31) = 2.372, *p* = 0.024, *d* = 0.42, but no significant difference was found for the control group, *t*(31) = 1.310, *p* = 0.200, *d* = 0.23.

### 3.5. Breathing Frequency_morning_

A repeated-measure ANOVA revealed no main effect of time, *F*(1,62) = 1.481, *p* = 0.228, partial *η*^2^ = 0.02, nor any interaction effect time × condition *F*(1,62) = 1.247, *p* = 0.268, partial *η*^2^ = 0.02.

### 3.6. Relationships between PSQI and CVA

Finally, a significant correlation was found between PSQI change and CVA_night_ change (*r* = −0.29, *p* = 0.018), while a tendency was found regarding the relationship between PSQI change and CVA_morning_ change (*r* = −0.24, *p* = 0.052). The negative correlation reflects the fact that a decrease in PSQI between pre-test and post-test is related to subjective sleep quality improvement, while an increase in CVA between pre-test and post-test reflects an improvement in CVA functioning.

## 4. Discussion

This study was aimed to investigate the influence of a 30-day slow-paced breathing intervention (experimental group) in comparison to social media use (control group) on subjective sleep quality via the PSQI and on night and morning CVA, operationalized via high-frequency heart rate variability. Consistent with our hypotheses, subjective sleep quality and CVA_night_ was increased in the experimental group but not in the control group, while there was only a tendency for CVA_morning_ to display a pattern similar to CVA_night_.

Regarding subjective sleep quality, confirming our hypothesis, scores on the PSQI significantly decreased for the experimental group between pre-test and post-test, which reflects a better subjective sleep quality in the group performing slow-paced breathing. This is in line with the expected relaxing effects of slow-paced breathing [10,12,20] and also with findings demonstrating that slow-paced breathing can decrease subjective feelings of anxiety [31]. Finally, this result is in line with the effects of slow-paced breathing observed on objective sleep parameters in insomnia patients [42].

Regarding CVA_night_, confirming our hypothesis, there was a significant increase for the experimental group between pre-test and post-test, while no changes were found in breathing frequency, which means that CVA changes were not driven by changes in breathing frequency. This result is in line with the resonance model [12] arguing that slow-paced breathing increases vagal afferences, and this is also in line with previous research showing that slow-paced breathing increases vagal efferences, and specifically CVA [16,31]. Following Werner and colleagues [37], we do not argue that CVA_night_ represents an indicator of sleep quality; however, we suggest that CVA_night_ may still reflect some form of cardiovascular self-regulation and recovery processes based on the restorative function of the parasympathetic nervous system [15], given during sleep the organism is much less under the influence of external factors. This view is also supported by previous research showing a lower CVA_night_ in individuals with sleep disorders [32,33,34], and also by research showing an association between higher CVA_night_ and higher subjective sleep quality [34,35,36]. Finally, this is also complemented by our findings concerning the significant relationship observed between PSQI change and CVA_night_ change (and the tendency observed with CVA_morning_ change), reflecting an association between improvement in CVA functioning and improvement in subjective sleep quality.

Regarding CVA_morning_, our hypothesis is partially validated, given it displayed only marginally a similar pattern to CVA_night_, meaning there was a tendency (*p* = 0.024) in the experimental group to display an increase in CVA_morning_ at post-test in comparison to pre-test, the effect size being lower for CVA_morning_ (*d* = 0.42) in comparison to CVA_night_ (*d* = 0.68). While the arguments already mentioned for CVA_night_ may also apply here, other processes may also be involved. More specifically, it has been suggested that the role of CVA during wake periods is more relevant than during sleep given a higher solicitation of self-regulation processes when the organism is awake [37]. 

The main strength of our study is the long-term (30 days) slow-paced breathing intervention, given most of the studies take only into account the short-term effects of slow-paced breathing on heart rate variability (e.g., [16,31,42]). Nonetheless, our study had some limitations. The main one is that the investigation of slow-paced breathing on sleep quality would require the use of the gold standard, polysomnography [46], as was done by Werner and colleagues [37]. However, it should still be mentioned that there is no established definition for objective sleep quality, and that sleep quality can refer to different variables measured with polysomnography [47]. Further, investigating separately the sleep stages seem also required, given the differential activation of CVA in REM and non-REM sleep [38]. Particularly, investigating CVA during slow wave sleep appears promising [39,48,49,50], given slow wave sleep is (mostly) free of any external confounding events, and is characterized by fewer body movements or arousals that provoke disruptions in the ECG signal, therefore ensuring higher stationarity of the ECG signal. Although some algorithms are being developed to identify sleep stages directly via heart rate variability measurements [49,50], preliminary research testing the influence of slow-paced breathing on sleep quality should definitely consider the use of polysomnography [37,46]. Another limitation is that we did not control for smartphone use before sleeping prior to the experiment. Moreover, we had no control group with paced breathing instructions, like in the study by Tsai et al. [42] where the control condition involved breathing at 12 cpm (however, the inhalation and exhalation phases were not specified). Further related to the control group, social media use has been found to decrease sleep quality in adolescents and young adults [51,52,53]. This was not found in our study—in our sample both PSQI scores and CVA values did not change between pre-test and post-test for the control group. This may potentially be because our participants already had habitual use of social media prior to sleeping before being recruited for the experiment. Further research may investigate alternative active control groups, as mentioned above with the 12 cpm breathing condition [42]. Finally, the present study tested young healthy individuals, and the findings cannot be generalized to other populations.

## 5. Conclusions

In summary, this study was aimed to investigate the effects of a smartphone-based slow-paced breathing intervention (6 cpm) performed for a duration of 15 min before sleeping across 30 days, compared to a control condition with participants using social media on their smartphone. Results showed that in the experimental group subjective sleep quality was improved and CVA_night_ was increased, while a marginal increase was also found in CVA_morning_. Taken together, our results suggest that slow-paced breathing performed before sleeping may enhance restorative processes at the cardiovascular level during sleep. Future research should investigate the effects of slow-paced breathing on sleep via polysomnography.

## Figures and Tables

**Table 1:** Descriptive statistics for subjective variables.

	Pre-Test				Post-Test			
	Experimental		Control		Experimental		Control	
	*M*	*SD*	*M*	*SD*	*M*	*SD*	*M*	*SD*
Time spent in bed	447.9	55.37	450.91	60.18	446.38	55.28	447.66	58.63
PSQI	3.31	1.20	3.44	1.01	2.91	1.38	3.75	1.34

Note: PSQI = Pittsburgh Sleep Quality Index (a lower score indicates a better sleep quality).

**Table 2:** Descriptive statistics for heart rate variability parameters

	Pre-Test								Post-Test							
	Experimental				Control				Experimental				Control			
	Morning		Night		Morning		Night		Morning		Night		Morning		Night	
	*M*	*SD*	*M*	*SD*	*M*	*SD*	*M*	*SD*	*M*	*SD*	*M*	*SD*	*M*	*SD*	*M*	*SD*
Interval R-R	1133.21	188.84	1128.98	151.47	1095.92	185.75	1098.94	120.37	1156.38	182.88	1160.48	177.01	1061.72	148.61	1072.42	133.96
SDNN	123.48	64.87	147.31	49.99	129.65	56.88	160.70	45.68	128.27	55.05	152.98	46.83	111.92	51.47	152.46	43.09
Heart rate	55.22	8.64	55.20	6.97	57.63	11.02	56.72	6.04	54.18	9.00	54.08	8.27	58.46	8.08	58.34	7.88
RMSSD	80.93	34.41	88.14	41.22	92.84	42.61	91.46	37.75	100.38	42.44	104.13	45.77	84.18	43.53	83.30	32.86
pnn50	42.49	19.57	44.52	21.48	48.14	20.04	44.98	16.69	49.79	23.05	50.19	20.62	41.07	18.66	41.62	15.72
LF (FFT) ms2	2856.38	2712.27	3136.33	1839.50	3688.52	3036.68	3518.83	1883.52	2888.49	2014.11	3619.39	1996.06	3538.59	2993.11	3290.56	1818.72
HF (FFT) ms2	2158.46	1511.91	2403.97	1751.62	2570.33	1558.90	2523.39	1491.46	2991.32	2234.31	3102.83	1932.98	2184.39	1816.48	2258.43	1448.96
LF/HF (FFT)	1.65	1.28	1.68	0.87	1.84	2.02	1.62	0.81	1.44	1.25	1.37	0.72	1.96	1.40	1.69	0.80
LF (AR) ms2	3161.06	2214.22	2955.02	1737.62	3860.79	3213.23	3241.82	1782.90	3186.44	2049.94	3433.10	1915.51	3261.93	2336.55	3042.67	1727.27
HF (AR) ms2	2257.52	1786.46	2712.10	2361.83	3386.60	3508.39	3058.16	2791.85	3620.01	3101.24	3699.39	2959.98	2801.72	3136.46	2578.36	2409.52
LF/HF (AR)	1.83	1.21	1.55	0.85	1.66	1.22	1.42	0.64	1.38	0.96	1.26	0.66	1.72	1.22	1.48	0.65
Breathing frequency	14.28	2.27	14.16	2.14	13.31	2.24	13.60	1.75	13.66	2.19	13.83	2.03	13.26	2.07	13.84	1.78

Note: SDNN = standard deviation of NN intervals, RMSSD = root mean square of successive RR interval differences, pNN50 = percentage of successive RR intervals that differ by more than 50 ms, LF = low-frequency, HF = high-frequency, FFT = fast Fourier transform, AR = autoregressive model.

## Supplementary Materials

The following are available online at http://www.mdpi.com/2077-0383/8/2/193/s1, Table S1: Full dataset.
